# Webb Miller and Trey Ideker To Receive Top International Bioinformatics Awards for 2009 from the International Society for Computational Biology

**DOI:** 10.1371/journal.pcbi.1000375

**Published:** 2009-04-24

**Authors:** B. J. Morrison McKay, Clare Sansom

**Affiliations:** 1 International Society for Computational Biology, University of California San Diego, La Jolla, California, United States of America; 2 Birkbeck College, London, United Kingdom

Each year, the International Society for Computational Biology (ISCB; http://www.iscb.org) makes two major awards to recognize excellence in the field of bioinformatics. The ISCB Awards Committee, composed of current and past directors of the Society and previous award winners, has announced that the 2009 ISCB Accomplishment by a Senior Scientist Award will be given to Webb Miller (Image 1) of The Pennsylvania State University, University Park, Pennsylvania, United States of America (USA), and the 2009 ISCB Overton Prize for outstanding achievement in early- to mid-career will be awarded to Trey Ideker (Image 2) of the University of California San Diego, La Jolla, California, USA, who serves on the Editorial Advisory Board for *PLoS Computational Biology*. These awards represent the highest tribute and recognition of scientific excellence within the bioinformatics community, and they are seen as honors well beyond the boundaries of the discipline. Both 2009 award winners started their careers in basic computer science, but since moving into bioinformatics they have made important contributions to biological understanding as well as to algorithm design.

**Image 1:**
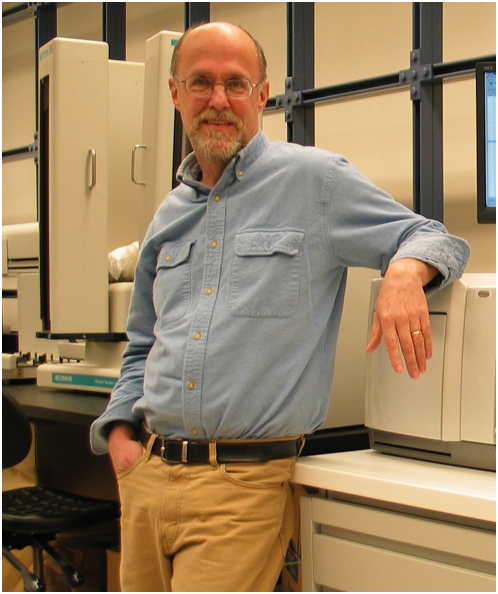
Webb Miller. Image credit: Lynn Tomsho, Pennsylvania State University

**Figure:**
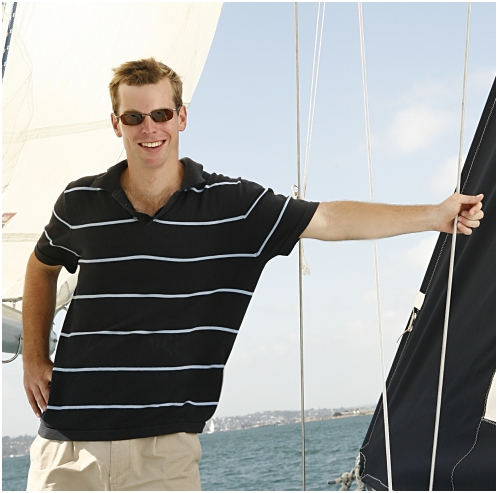
Trey Ideker. Image credit: Jason Varney, Jason Varney Photography

Both awards will be presented at the Society's flagship international conference, Intelligent Systems for Molecular Biology (ISMB), where the winners will give keynote presentations. In 2009, the (seventeenth) annual ISMB will be held in conjunction with the European Conference on Computational Biology (ECCB) for the third time; ISMB/ECCB 2009 will take place in Stockholm, Sweden, from June 27 through July 2.

## Accomplishment by a Senior Scientist Award: Webb Miller

Established in 2003, ISCB's Accomplishment by a Senior Scientist Award recognizes members of the computational biology community who have made major contributions to the field through research, education, service, or a combination of the three. Miller will be joining a prestigious group of previous winners: David Sankoff (University of Ottawa, Canada), David Lipman (US National Center for Biotechnology Information, USA), Janet Thornton (European Bioinformatics Institute, United Kingdom (UK)), Mike Waterman (University of Southern California, USA), Temple Smith (Boston University, USA), and David Haussler (University of California Santa Cruz, USA).

Ten years ago, the name Webb Miller was already well-known to bioinformaticians worldwide for two very highly cited classic papers on the BLAST algorithms for searching sequence databases [1],[2]. In recognizing an author of a widely used algorithm with the Senior Scientist Award, ISCB is continuing a tradition: the three most recent winners of this Award, David Haussler, and Temple Smith and Michael Waterman, are associated, respectively, with Hidden Markov Models and the eponymous Smith–Waterman sequence alignment algorithm. Today, however, Miller's name is equally well-known for the alignment, comparison, and analysis of complete vertebrate genomes. Much of the code written in his group is embedded in the University of California Santa Cruz (UCSC) Genome Browser, through which these algorithms penetrate as widely into the bioinformatics and genomics community as the ever-popular BLAST.

Miller's initial training was, like Haussler's, in mathematics. In the mid-1960s, when still an undergraduate student at Whitman College in Walla Walla, Washington, he found a book in the library that determined the path of his higher education and career for the next two decades. It was on the theoretical limits of what is computable, and the young Miller immediately decided that he, at his “little college”, could undertake real, publishable research in this field. The resulting paper, the first of a very long list, eventually appeared in an obscure logic journal published in East Germany. This led him to graduate work at Whitman in Computer Science, to his Ph.D. in Mathematics at the University of Washington Seattle, and, by 1969, to an assistant professorship in Computer Science at The Pennsylvania State University (Penn State). His work at this time was still focused on theoretical computer science; even when he started at Penn State he had no experience of practical computing or writing code.

In 1980, after a decade or so working on the fundamental issues behind how computers deal with floating-point numbers, Miller took a break from research to write three books, including a software engineering textbook and a handbook of software tools. While he was writing, he was also looking around for new challenges and applications of his computational knowledge. He found them through a most unexpected source. “My mother started sending me newspaper clippings about the beginnings of the Human Genome Project,” he says. “This fascinated me, although I knew no biology at the time. I hadn't even taken it in high school.” During the next few years, he worked at IBM, the University of California Santa Barbara, and the University of Arizona. By the time he returned to Penn State as a full professor of computer science in 1987, his research was focusing wholly on computational biology.

Miller's first important collaborator in bioinformatics was Gene Myers, whom he met while at the University of Arizona and whom he still cites as one of the greatest influences on his scientific career. During the 1987–1988 academic year, Myers came to Penn State for sabbatical, for research, and to teach advanced courses on DNA sequence analysis. Miller's collaboration with Myers and with David Lipman, director of the newly founded National Center for Biotechnology Information in Bethesda, Maryland, led to the development of the BLAST algorithm. Lipman, who received the ISCB Senior Scientist Accomplishment Award in 2004, was already well-known for the FastA algorithm; BLAST emerged from a search for a similar sequence comparison algorithm that would be fast enough to make database searching feasible.

Soon after entering bioinformatics, Miller turned his attention from general sequence alignment algorithms to the specific problem of aligning long DNA sequences. “Most bioinformaticians spent the 1990s waiting for the human genome sequence,” he said. “My question was: How soon would the second vertebrate genome come out, so I could try a genome-wide sequence alignment?” That second genome—of the mouse—was published in 2002 [3]. “I originally anticipated that we would have two vertebrate genomes by the time I reached retirement age in 2008. Instead, thanks to improvements in sequencing technology, we now have over forty.” An alignment of forty-four vertebrate genomes has recently been released on the UCSC Genome Browser. Haussler collaborated with Miller closely throughout the development of the Genome Browser, and he greatly values him as a colleague as well as for his vital contribution to the field of comparative genomics. “Webb has played an essential role in nearly every vertebrate genome sequence project. He developed the first program capable of accurate comparative alignment for entire vertebrate-sized genomes,” he says.

Miller has never been particularly interested in the properties of protein-coding genes, preferring to study the biology of functional, non-coding regions of DNA, such as regulatory regions. And here he confesses to some disappointment with the rate of progress. “It has been easier to sequence genomes, but harder to understand their biology than I, coming from a computer science background, thought it would [be],” he says. “It seems that biology is even more complex than we had assumed.”

He and his collaborators have now taken on a new challenge: sequencing the genomes and understanding the biology of rare, endangered, and even extinct species. He has published sequences of the nuclear genome of the woolly mammoth [4] and the mitochondrial genome of the Tasmanian tiger (*Thylacinus cynocephalus*) [5], which became extinct in 1936. The DNA samples for the latter project were obtained from specimens that had been preserved for decades in museums. Miller says he is hoping that similar sequencing techniques will help preserve endangered species from extinction. One of these is the so-called Tasmanian devil, a ferocious marsupial that is now under threat from a mysterious, contagious tumor: Devil Facial Tumor Disease (DFTD). “We are sequencing two specimens, one with the disease and another that seems immune, and hope to use the differences to guide a breeding program,” he says.

Miller acknowledges that he owes much of his success to “great” collaborators, from Myers and Lipman in the late 1980s to Haussler and his UCSC colleagues Jim Kent and Tom Pringle. And it may be that great collaborators make each other. “Time and time again, Webb has made major contributions and taken little credit for himself, preferring to put younger researchers in the limelight, whether or not they were his students. I've never worked with a more generous collaborator,” says Haussler.

## Overton Prize: Trey Ideker

The ISCB Overton Prize was established in 2001 in memory of G. Christian Overton, a major contributor to the field of bioinformatics and a member of the ISCB Board of Directors who died suddenly the previous year. The Prize is awarded for outstanding accomplishment to a scientist in early- to mid-career who has already made a significant contribution to the field of computational biology. Previous recipients are Christopher Burge (Massachusetts Institute of Technology, USA), David Baker (University of Washington, USA), W. James Kent (University of California Santa Cruz, USA), Uri Alon (Weizmann Institute of Science, Israel), Ewan Birney (European Bioinformatics Institute, UK), Mathieu Blanchette (McGill University, Canada), Eran Segal (Weizmann Institute of Science, Israel), and Aviv Regev (The Broad Institute of Harvard University and Massachusetts Institute of Technology, USA).

Trey Ideker, winner of the 2009 ISCB Overton Prize, was, like Miller, initially trained in computer science. His decision to move into bioinformatics for his graduate studies was taken for more ideological than scientific reasons. “I had been working for the defense industry as a college student, and then in my first professional job, and while it is fascinating to model the movement of flying objects, I didn't want to spend my career making bombs. I therefore started looking for alternative ways to use my computing knowledge, including in molecular biology,” recalls Ideker. A friend who was a molecular biologist in graduate school gave him the names of potential advisors, one of whom was Leroy Hood, co-founder of the Institute of Systems Biology (ISB) in Seattle, Washington. In 1996, and before the ISB was founded, Ideker moved to Seattle to join Hood as one of his first graduate students there. This move set the stage for a career that has already produced some groundbreaking work in the area of network biology.

Ideker's graduate studies coincided with the later years of the Human Genome Project. “When I joined the lab, everyone there was working on sequence analysis,” he remembers. “I was working with a talented physician and postdoc, Pete Nelson, who was studying the pathways in cells that led to the development of prostate cancer. I started trying to model these pathways; this project fell on its face, as it was far too complex for a single graduate student, but the ideas it generated have become the basis for my whole career.” Working closely with Hood, he—while still a student—was one of the first to publish an integrated computational model of a metabolic network; this paper [6], published in 2001, has already been cited more than 850 times. With this very promising start, he understandably had many offers of positions as an independent group leader. He initially moved to the prestigious Whitehead Institute for Biomedical Research in Cambridge, Massachusetts, before family drew him back to the West Coast. He is now settled at the University of California San Diego (UCSD) as an associate professor. “UCSD is a fantastic place to do science. I can't imagine being more productive anywhere else. The growth of biotechnology in the San Diego area in recent years has been overwhelming.”

While at the Whitehead Institute and in collaboration with others looking at similar problems, Ideker further developed a prototype network modelling program that he had worked on in Hood's lab into what is now the widely used online tool Cytoscape [7]. “We decided to pool our ideas and resources rather than reinventing the wheel,” he says. Cytoscape is now freely available under an open source license, and this has attracted a far larger pool of both users and developers than would have been attracted to a commercial enterprise.

Ideker continues to be involved in the Cytoscape project, and since moving to San Diego his research has become focused on comparing networks between and within species. This work has already made important contributions beyond systems biology and has convinced Aviv Regev, his immediate predecessor as winner of the ISCB Overton Prize, of its significance. “Trey's work has epitomized the power of integrating innovative computational methods with cutting-edge genomics. His pioneering work has set a model for doing systems biology that has been followed by numerous groups and has impacts for understanding the evolution of biological systems and for treating disease.”

In the 1990s, bioinformatics was dominated by DNA and protein sequences, with about 95% of its effort going into sequence mining and comparison. Ideker believes that networks are at the next level of complexity from sequences and that similar techniques can and should be used for analyzing them. “It was obvious to me that we needed a searching and comparison tool that would be something like BLAST for networks,” he says. The PathBLAST program [8] does just that, and the ideas behind it have spawned a number of similar tools.

More recently, Ideker has been turning his attention to medical applications of network modeling. He has compared the complete map of protein–protein interactions for the malaria parasite *Plasmodium falciparum*, generated using the yeast-two-hybrid method, against other eukaryotic networks, and proposed unique features of its metabolism that might be targeted in designing drugs against this destructive disease [9]. He has also showed that grouping proteins into pathways and taking the average of the levels of each protein in a single pathway can add 8%–9% to the accuracy of prognostic predictions in breast cancer [10]. And he is about to take his involvement in medical applications to a new level, as, in mid-2009, he takes up the position of head of genetics at a new institute within the University of California San Diego School of Medicine. “I will be moving from the periphery into the center of genomics-based medical research,” he says. “My vision is to integrate network analysis into medicine and develop useful clinical tools.”

Ideker's career has already been marked by conspicuous success, and he is well-placed to succeed in this next challenge as well. He adds his Overton Prize to a list of honors that includes recognition by MIT's *Technology Review* as one of the top ten innovators of 2006. The ISCB Awards Committee members couldn't agree more.

## Additional Information

These ISCB award winners will be joined by six other distinguished keynote speakers at the ISMB/ECCB 2009 conference in Stockholm, Sweden, in late June. The conference will also feature half-day special sessions on emerging topics, full-day special interest group meetings, highlights presentations of research published during the previous year with recent updates, technology demonstrations and workshops, poster sessions, an art-in-science exhibition, vendor exhibits, and several networking events. The conference organizers anticipate an attendance of more than 1,400 scientists and are currently finalizing an agenda that is expected to offer more than 150 oral presentations. For the full conference program and registration information, please visit http://www.iscb.org/ismbeccb2009/index.php.

To read more about each of the past winners of ISCB's Accomplishment by a Senior Scientist Award and Overton Prize, please see http://www.iscb.org/iscb-awards.
